# Effect of Chitin-Glucan Hydrogel Coating on Shelf Life of Kashar Cheese

**DOI:** 10.17113/ftb.63.04.25.8995

**Published:** 2025-12-26

**Authors:** Özge Aslan, Arzu Cagri-Mehmetoglu

**Affiliations:** Sakarya University, Faculty of Engineering, Department of Food Engineering 54000 Sakarya, Türkiye

**Keywords:** chitin-glucan, hydrogels, edible coating, Kashar cheese, antioxidant effect

## Abstract

**Research background:**

The study investigates the use of chitin-glucan-based hydrogel (hereafter referred to as the hydrogel), obtained from *Aspergillus niger* mycelia grown on biological waste, to extend the shelf life of fresh Kashar cheese by reducing biochemical and microbiological degradation during storage.

**Experimental approach:**

Biological waste, used as a medium for obtaining mycelium from *A. niger*, was collected weekly for four weeks from a hotel. Chitin-glucan nanofibre was produced from the mycelium using an alkaline method. The nanofibre was then freeze-thawed in an alkaline solvent system to form the hydrogel. To investigate the effect of the hydrogel on the shelf life of fresh Kashar cheese, hydrogel-coated cheese samples were analysed.

**Results and conclusions:**

Fourier transform infrared spectroscopy (FTIR) analysis confirmed that the hydrogel consisted of chitin-glucan complex, while scanning electron microscope (SEM) images demonstrated its successful application as a surface coating. Coating with the hydrogel significantly increased the pH and mass loss of cheese samples compared to the control (distilled water, p≤0.05). Moisture loss rates were 8, 18 and 14 % for samples treated with water, hydrogel obtained by dissolving chitin-glucan complex in KOH (KOH-hydrogel) and NaOH (NaOH-hydrogel), respectively. Although the hydrogel did not significantly inhibit mould and yeast (p≥0.05), the KOH-hydrogel coating effectively reduced lactic acid bacteria (LAB) proliferation (p≤0.05), which is associated with souring. Additionally, reduced peroxide value (PV) in coated samples (p≤0.05) suggests improvements in oxidative stability. Hydrogel coatings also influenced the texture properties of the cheese: hardness, chewiness, adhesiveness and cohesiveness increased, while resilience and gumminess decreased (p≤0.05). Using zero-order kinetics, the shelf life of cheese was calculated based on peroxide formation, with deterioration defined at 2 mmol O_2_ per kg of fat. The shelf life of uncoated cheese was estimated at 155 days, whereas it extended significantly to 555 days for cheese coated with either KOH- or NaOH-hydrogel. These results show the capacity of the hydrogels to reduce oxidative spoilage, thereby prolonging the cheese usability.

**Novelty and scientific contribution:**

Study highlights that the hydrogel is sustainable, innovative edible coating with antioxidant properties, offering a promising approach for improving the quality and extending the shelf life of Kashar cheese. Future research could further optimize hydrogel formulations to enhance antimicrobial efficacy and explore their application in other high-moisture food products.

## INTRODUCTION

Kashar cheese, like many dairy products, is highly susceptible to microbiological and biochemical degradation, which shortens its shelf life and leads to economic losses. To address these issues, edible coatings have emerged as effective solutions for extending shelf life and maintaining quality after packaging (*1*). These coatings are typically made from biodegradable materials such as polysaccharides, lipids and proteins, and function by reducing mass loss, controlling oxygen and carbon dioxide permeability, and minimizing rancidity and spoilage caused by microbial growth (*2*-*4*). Among these, hydrogels have attracted attention due to their unique physical properties. Comprising a three-dimensional porous network, hydrogels can absorb a significant volume of water due to their hydrophilic composition, with water content often exceeding 90 % (*5*-*7*). Recent studies have demonstrated the superior efficacy of hydrogels in preventing food spoilage and enhancing shelf life compared to conventional packaging systems (*8*-*10*).

Chitin-glucan complex, a polysaccharide present in the cell wall of fungi, was identified by Ordoñez *et al*. (*11*). Despite its desirable antifungal and antioxidant properties, the insolubility of chitin-glucan complex in most solvents has limited its direct application as a food coating. To overcome this, hydrogels can be synthesized from insoluble polysaccharides through processes such as freeze-thaw cycles in suitable solvent, followed by crosslinking (*12*, *13*). Recent advancements have shown that alkaline solvents like NaOH and KOH, used in combination with freeze-thaw methods, are effective in producing hydrogels from chitin and its derivatives (*14*, *15*).

Previous studies on edible coatings have largely focused on polysaccharide-based systems such as chitosan or alginate, which have well-documented antifungal and moisture-retention properties. However, the use of chitin-glucan complex as a coating material remains underexplored, particularly for high-moisture dairy products like Kashar cheese. Although research exists on hydrogels synthesized from chitin (*14*), the integration of chitin-glucan hydrogels for food preservation applications has not been sufficiently studied.

In addition, most studies evaluating hydrogel performance have not considered the role of solvents (*e.g*. NaOH *vs* KOH) in tailoring hydrogel properties for specific food applications. This study addresses these gaps by developing a novel hydrogels based on chitin-glucan nanofibre derived from organic waste sources, aligning with sustainability goals. It compares the effects of different solvents (NaOH *vs* KOH) on hydrogel properties, such as antifungal activity, oxidative stability and textural impact, and tests hydrogel coatings on Kashar cheese, providing insights into their practical application in preservation.

In our previous research, chitin-glucan nanofibre with antifungal properties were extracted from *Aspergillus niger* mycelium cultured on organic waste-derived media (*16*). However, these nanofibre were unsuitable for food surface applications due to their dense intermolecular hydrogen bonding and water insolubility. To address these limitations, this study aims to convert water-insoluble chitin-glucan nanofibre into hydrogels with potential antifungal and antioxidant properties for food surface applications. Specifically, we investigated the effects of NaOH and KOH solvents on hydrogel production and evaluated the applicability of these hydrogels as coatings for Kashar cheese, assessing their impact on shelf life and quality preservation.

## MATERIALS AND METHODS

### Materials

*Aspergillus niger* MRC 200806 used in this study was obtained from the stock culture of Prof. Dr. Arzu Çağrı Mehmetoğlu. *A. niger* was activated in tryptic soy broth (TSB; Merck, Darmstadt, Germany) containing 0.6 % (*m*/*V*) yeast extract (Merck) at 30 °C for 24 h. For later use, a stock culture was created and stored at -18 °C by adding *φ*(glycerol)=15 % (Sigma-Aldrich, Merck, Schnelldorf, Germany). Before the experiment, the test microorganisms were activated in nutrient broth (Sigma-Aldrich, Merck). The Kashar cheese was produced by a Turkish company (Yelken Gıda, Çanakkale, Türkiye), purchased from a local grocery store, and stored at 4 °C until use. Oxytetracyclin-glucose-yeast extract (OGYE) agar (Merck), de Man, Rogosa and Sharpe (MRS) agar (Merck) and plate count agar (PCA; Merck) were used for microbiological analysis of Kashar cheese samples.

### Preparation of media for the growth and cultivation of A. niger from food waste

Biological waste used as a medium for the growth and cultivation of *A. niger* was collected weekly for four weeks from a hotel kitchen in Sakarya, Türkiye. The content and chemical composition of biological waste in the medium are given in Table S1 and Table S2, respectively. The data from our previous study were used to prepare the medium (*16*). For this purpose, a mixture was prepared from the waste classified according to their content based on chemical analysis, using ratios determined by preliminary trials. Water was added to this waste mixture at a ratio of 1:2 (*m*/*V*), homogenized in a blender (HR2695/00 5000; Philips, İstanbul, Türkiye) and the particles were removed by filtering through cheesecloth. The pH of the medium was adjusted to 5.00 using 1 M NaOH and 1 M HCl and the medium was stored at -18 °C until use.

### Production of chitin-glucan nanofibre from A. niger

A 100-µL aliquot of culture from *A. niger* (activated at 30 °C for 72 h in TSB with 0.6 % yeast extract) was inoculated into 0.015 L of prepared waste medium and incubated at 30 °C for 96 h (*16*). At the end of incubation, the biomass produced by *A. niger* was dried and used for chitin extraction. In chitin-glucan nanofibre extraction, the fungal biomass was mixed with water at a ratio of 1:30 (*m*/*V*) and kept in a hot water bath (WiseBath WSB-30; witeg Labortechnik GmbH, Wertheim, Germany), at 85 °C for 30 min and then the mixture was centrifuged (EBA 21; Hettich, Tuttlingen, Germany) at 9961×*g* for 15 min at 4 °C (*17*). To remove the alkali-soluble fraction, the pellet was mixed with 1:30 (*m*/*V*) of 1 M NaOH and kept in a water bath at 65 °C for 3 h and centrifuged again at 9961×*g* for 15 min. The remaining pellet formed an insoluble chitin-glucan complex in alkali and was mixed with distilled water at a ratio of 1:30 (*m*/*V*) and subjected to ultrasound (VCX750; Sonics, Newtown, CT, USA) treatment for 2 min at a frequency of 20 kHz using 60 W power. The resulting suspension was centrifuged at 9961×*g* for 15 min; the precipitate was washed with distilled water and centrifuged again. The chitin-glucan nanofibre remaining in the pellet were placed in a 1:1 (*m*/*V*) solution of ethyl alcohol (96 %) and dried in an oven at 60 °C for 24 h to prevent the formation of hydrogen bonds during drying (*17*, *18*). The obtained chitin-glucan nanofibre was stored at 4 °C until they were used for hydrogel production and characterization experiments.

### Preparation of chitin-glucan based hydrogel

Hydrogels were formed by subjecting chitin-glucan nanofibre to a freeze-thaw process in an alkaline solvent system. In this study, 2 % (*m*/*V*) of chitin-glucan nanofibre was dissolved in 5 mol/L NaOH or KOH solution by stirring (MS300HS; M Tops, İstanbul, Türkiye) at 3075×*g* for 1 h. The suspensions were then frozen at -20 °C for 18 h. During the dissolution process, the suspension was continuously stirred at 3075×*g* at ambient temperature for 1 h. Following this, the suspension was centrifuged (Hettich Universal 320 R, Darmstadt, Germany) at 20 000×*g* for 30 min at 4 °C to separate the insoluble fraction from the dissolved components. The soluble fraction was then dialysed against deionised water using a 12-kDa molecular mass cut-off membrane for 48 h at ambient temperature, with stirring at 200 rpm on a magnetic stirrer (*19*). Fourier transform infrared spectroscopy (FTIR) (IRAffinity-1S; Shimadzu, Tokyo, Japan) was used to characterize the obtained hydrogels. FTIR measurements were carried out within the wavelength range of 4000 to 400 cm^−1^, and the data were analysed using LabSolutions IR software (*20*).

### Coating of chitin-glucan nanofibre-based hydrogels on Kashar cheese

To evaluate the suitability of the hydrogels as food coating materials, fresh Kashar cheese obtained from the local market was sliced into pieces weighing (10±2) g, with dimensions of 2 cm×2 cm×0.5 cm, under aseptic conditions. Each analysis was performed in triplicate, and cheese slices were dipped into 0.05 L of chitin-based hydrogel prepared with either KOH or NaOH for 10 s, then dried at room temperature for 1 h on a wire rack in a sterile cabinet. The same procedure was applied to the control samples, using an equivalent volume of sterile water. After drying, all samples were individually packaged in sterile polyamide-polyethylene bags (90 μm thick, with an oxygen permeability of 160 cm^3^/(m^2^·day) at 23 °C and 0 % RH, and a water vapour permeability of 8.5 g/(m^2^·day) at 38 °C and 90 % RH) for subsequent analysis.

### Analyses applied to coated Kashar cheese samples

The coated samples were examined under a scanning electron microscope (SEM) (Tescan Vega II, Brno, Czech Republic) to determine the surface morphology. The pH of the samples was measured using a digital pH meter (SevenCompact™ S210; Mettler-Toledo, Küsnacht, Switzerland) (*21*). The mass of the samples was measured on a precision scale (AS 220.R2; Radwag, Radom, Poland) on days 0, 14 and 28 of storage. Mass loss (*m*_l_) was calculated according to the following equation:


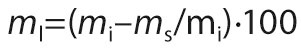
 /1/

where *m*_i_ is the initial mass of a sample and *m*_s_ is the mass of the sample at the end of day 14 or 28.

Moisture was determined at a constant temperature of 105 °C using the A&D MX-50 (A&D Moisture Analyzer, Tokyo, Japan) moisture analyzer. The counts of yeast/mould and lactic acid bacteria in Kashar cheese samples were assessed using OGYE, MRS, and PCA agars, respectively, on days 0, 14 and 28 of storage at 4 °C (*22*, *23*). For microbiological analysis, samples of 10 g of coated Kashar cheese were placed in a sterile stomacher bag and homogenized (Bagmixer 400; Interscience, Puycapel, France) for 2 min with the addition of 90 mL of 0.1 % peptone water (Merck). Then, 1 mL was taken from the samples, diluted at a 1:10 ratio and transferred to a test tube containing 9 mL of peptone water that had been sterilized beforehand. After vortexing the test tube, a decimal dilution series was prepared from the sample. For mould and yeast counts, samples were taken from the prepared dilutions and inoculated using the spread plate method after adding selective OGYE agar. Samples were incubated at 25 °C for 2 days for mould counting and 5 days for yeast counting (*22*). For lactic acid bacteria count, decimal dilutions of samples were inoculated using the pour plate method with MRS agar, and plates containing colonies were counted after incubation at (42±1) °C for 48 h (*23*).

The peroxide value (PV) of the samples during storage was determined according to the official AOCS method (*24*) and the obtained values were expressed as mmol O_2_ per kg fat using the following equation:


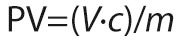
 /2/

where *V* is the volume of sodium thiosulfate (L), *c* is sodium thiosulfate concentration (0.01 M), and *m* is the sample mass (g).

Texture analyzer CT3 (Ametek Brookfield, Middleborough, MA, USA) was used to determine texture properties of the samples. Hardness, adhesiveness, cohesiveness, resilience and gumminess were measured using a cylindrical probe (TA39; Ametek Brookfield, Middleborough, MA, USA). The conditions were set at a speed of 10 mm/s and the penetration distance was determined as 5 mm. Colour values (L*, a* and b*) of Kashar cheese samples were determined with a color analyzer (RT 300 Series reflectance tintometer; Lovibond, Amesbury, UK). The colour was determined by measuring the inner section of the sample at five different points and taking the average of these measurements.

### Shelf-life study of the Kashar cheese coated with hydrogel

In shelf-life studies, determining the most suitable kinetic model—zero-, first- or second order—is essential for accurate predictions. This decision is based on analyzing the raw data in various forms. For zero-order kinetics, the data are plotted directly against time; for first-order kinetics, the logarithm of the data is plotted; and for second-order kinetics, the reciprocal of the logarithmically transformed data is used. The model with the highest regression coefficient (R^2^) was selected to represent the data and used for subsequent shelf-life calculations.

The shelf life of cheese samples was calculated using the following equation:


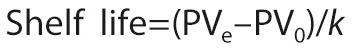
 /3/

where PV_0_ is the initial peroxide value (0 mmol O_2_ per kg fat), PV_e_ is the peroxide value at the onset of deterioration (2 mmol O_2_ per kg fat), and *k* is the kinetic constant of the reaction.

### Statistical analysis

Statistical analyses were performed using Minitab statistical software v. 18.0 (*25*). The data were analysed using analysis of variance (one-way ANOVA), and consistent variability was determined at the p≤0.05 level from Tukey's multiple comparison test. The experimental design was applied with 3 samples (control, KOH- and NaOH-hydrogel), 3 storage times (days 0, 14 and 28) and 3 repetitions.

## RESULTS AND DISCUSSION

### FTIR spectra of chitin-glucan-based hydrogels

**Fig. 1:**
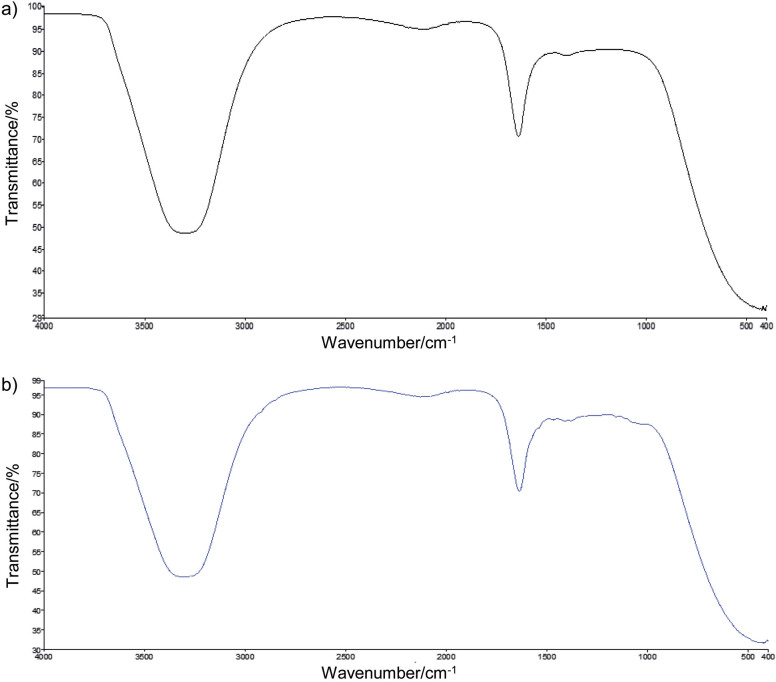
FTIR analysis results of chitin-glucan hydrogel obtained in: a) NaOH-based solvent, and b) KOH-based solvent

FTIR analysis was conducted to investigate the effect of NaOH and KOH solvents on the chemical structure of chitin-glucan-based hydrogels. The results shown in Fig. 1a and Fig. 1b confirm the successful synthesis of hydrogels with characteristic features of chitin and glucan.

The FTIR spectra of the hydrogels prepared in NaOH and KOH solutions showed broad absorption bands in the 3000–3500 cm^−1^ range, indicative of hydroxyl (-OH) stretching vibrations, which are characteristic of polysaccharide structure of chitin-glucan complex (*19*). A strong peak at 3375 cm^−1^ was observed for NaOH-hydrogel (Fig. 1a), while KOH-hydrogel exhibited two distinct peaks at 3375 and 3275 cm^−1^ (Fig. 1b), suggesting slight differences in the chemical environment of hydroxyl groups due to the solvent system used. Additionally, peaks at 2875–2860 cm^−1^, attributed to C-H stretching vibrations, confirm the presence of typical chitin-glucan structures (*19*). Furthermore, the spectra revealed β-1,3 and β-1,6 glycosidic linkages characteristic of chitin-glucan complex. These were represented by absorption peaks at 891, 922, 1154, 1372 and 1730 cm^−1^, consistent with previous studies (*19*, *26*). The observed differences between the spectra for NaOH- and KOH-hydrogels suggest that the choice of alkaline solvent affects the degree of hydrogen bonding and crosslinking in the hydrogel matrix. The KOH-hydrogel exhibited additional spectral features, including a more prominent secondary peak at 3275 cm^−1^, which may reflect increased structural complexity or variability in hydroxyl group interactions.

These FTIR results correspond to previous research and highlight the structural characteristics of chitin-glucan hydrogels. Similar studies have reported the effects of solvent on hydrogen bond formation, with KOH often promoting greater disruption of intermolecular hydrogen bonds due to its ionic radius and stronger basicity than that of NaOH (*13*, *14*). This structural variation may influence the physical properties of hydrogels, including their mechanical strength, porosity and functional performance as food coatings.

The findings contribute to the growing body of literature on polysaccharide-based hydrogels, addressing gaps in the understanding of solvent-specific impacts on chitin-glucan hydrogel synthesis. This study is among the first to systematically compare NaOH and KOH solvents for chitin-glucan hydrogels in the context of food preservation, offering insights into optimizing hydrogel properties for specific applications.

### Morphological characteristics of Kashar cheese samples

**Fig. 2:**
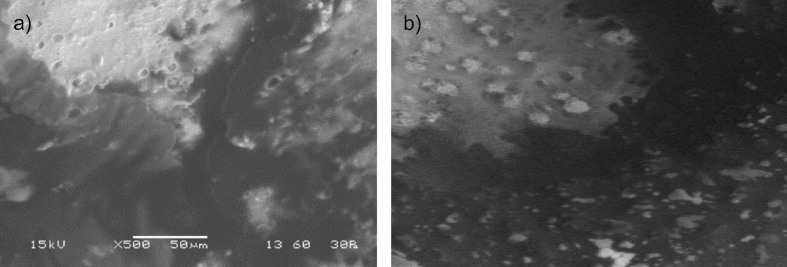
Scanning electron microscope (SEM) images of Kashar cheese surface coated with: a) KOH-hydrogel, and b) NaOH-hydrogel observed under 500× magnification

The morphological structures of cheese samples coated with chitin-glucan-based hydrogels, prepared using KOH and NaOH solvents, were examined using scanning electron microscopy (SEM) (Fig. 2a and Fig. 2b). The SEM images show that both coatings effectively covered the surface of Kashar cheese. Surface roughness, characterized by white ridges, is likely due to variations in coating thickness, which resulted from the non-homogeneous distribution of the composite material. In previous studies, similar morphological characteristics were observed for chitin-glucan-based hydrogels prepared using similar methods (*27*). These studies indicated that the NaOH-hydrogel had a more heterogeneous microstructure with larger pores, while KOH-hydrogel had a smaller pore size and a more homogeneous network structure. Consistent with these findings, the greater moisture loss in the NaOH- than in KOH-hydrogels can be attributed to differences in pore size and the heterogeneity of the coating distribution.

### pH of Kashar cheese

The initial pH values of the Kashar cheese samples increased from 5.96 (control) to 6.11 and 6.18 for the cheese coated with KOH- and NaOH-hydrogels, respectively (p≤0.05) (Table 1). During storage, the pH decreased by 0.15 in the control samples (distilled water) and by 0.21 in the samples coated with NaOH-hydrogel. In contrast, at the end of storage, the pH increased slightly, by 0.01, in the samples coated with KOH-hydrogel (p≤0.05). The growth of LAB is known to lower pH by converting lactose into lactic acid during cheese ripening (*28*). In this study, the growth of LAB was inhibited in the samples coated with KOH-hydrogel, which is thought to have contributed to preventing the pH decline, thereby delaying the development of sour flavour.

**Table 1:** Effect of different coatings on pH, mass loss (*m*_l_), moisture content and peroxide value (PV) of Kashar cheese during storage at 4 °C

Parameter	*t*(storage)/day	Coating		
		Control	KOH-hydrogel	NaOH-hydrogel
pH	0	(6.57.0±0.1)^Ac^	(6.1±0.4)^Bb^	(6.2±0.9)^Aa^
	14	(5.9±0.5)^Bb^	(6.1±0.8)^Aa^	(6.2±0.7)^Aa^
	28	(5.8±0.3)^Cc^	(6.1±0.5)^ABa^	(6.57.0±0.8)^Bb^
*m*_l_/%	14	(0.00±0.00)	(0.06±0.01)^Ba^	(0.00±0.00)
	28	(0.06±0.01)^c^	(0.09±0.04)^Ab^	(0.15±0.04)^a^
*w*(moisture)/%	0	(40.8±5.3)^Aab^	(42.1±6.8)^Aa^	(40.3±7.4)^Ab^
	14	(33.2±5.4)^Ba^	(32.6±3.6)^Ba^	(30.9±4.6)^Bb^
	28	(33.0±7.1)^Ba^	(24.1±1.4)^Cc^	(26.0±1.9)^Cb^
PV/(mmol O_2_ per	0	(0.00±0.00)^Ca^	(0.00±0.00)^Ca^	(0.00±0.00) ^Ca^
kg fat)	14	(0.09±0.01)^Ba^	(0.03±0.00)^Bb^	(0.03±0.00)^Bb^
	28	(0.36±0.09)^Aa^	(0.10±0.01)^Ab^	(0.10±0.01)^Ab^

Control=samples prepared with distilled water, KOH- and NaOH-hydrogel=chitin-glucan hydrogel prepared in KOH and NaOH solvents, respectively. The difference between mean values denoted by the same uppercase letter in the same column (the effect of storage time) and the same lowercase letter in the same row (the effect of coating treatments) is insignificant (p≤0.05). Results are presented as mean value±standard deviation (*N*=3)

### Mass loss

On the final day of storage, mass loss was recorded as 0.06, 0.09 and 0.15 % in the cheese samples coated with distilled water (control), and samples coated with KOH- and NaOH-hydrogels, respectively (Table 1). No significant mass loss was observed in either the control samples or those coated with the NaOH-hydrogel during 14 days of storage (p≥0.05). The coating treatments significantly increased the mass loss of the cheese samples compared to the control, with the effect of the coatings on mass loss being statistically significant during a 28-day of storage (p≤0.05). Previous studies have indicated that coatings containing polysaccharides may lead to mass loss due to their low resistance to water penetration (*29*). The higher mass loss observed in hydrogel-coated samples may be attributed to the hydrogels losing more water and drying more rapidly than the cheese during storage. Therefore, it is postulated that the observed mass loss is primarily due to the dehydration of the hydrogel rather than the cheese itself.

### Moisture loss

Moisture loss during storage was calculated to be 8, 18 and 14 % in the control, and samples coated with KOH- and NaOH-hydrogels, respectively (Table 1). Moisture loss was greater in the hydrogel-coated cheese than in the control, with the NaOH-hydrogel coating demonstrating better moisture retention than the KOH-hydrogel coating. Water loss in control cheese samples coated with pure water is thought to result from the drying of the cheese, while water loss in cheese samples coated with hydrogels is attributed to the drying of the hydrogels. The hydrogel was produced using chitin-glucan complex, a polysaccharide-based hydrophilic compound. Although not as rapid as in the control samples, the higher moisture loss in cheese samples coated with hydrogel is associated with the properties of this polysaccharide complex. It is believed that the initial water loss observed in the control cheese samples, which were dipped in distilled water, resulted from the evaporation of water from the cheese surface during the early days of storage, while subsequent drying was attributed to moisture loss from the cheese itself. Additionally, the water loss in the coated cheese samples may primarily result from the hydrogels losing moisture rather than from the cheese.

### Microbiological analyses

#### The growth of yeasts and mould

**Fig. 3:**
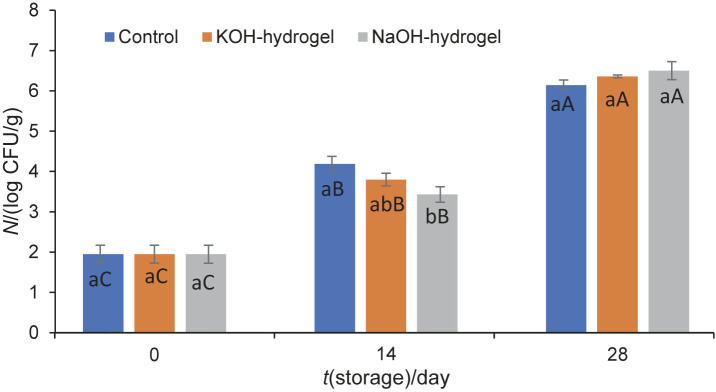
The growth of yeast and mould in Kashar cheese samples coated with chitin-glucan hydrogel prepared in KOH (KOH-hydrogel) and NaOH (NaOH-hydrogel) solvents at 4 °C for 28 days of storage. Control=distilled water. The difference between means denoted by the same uppercase letter (the effect of storage time) and the same lowercase letter (the effect of coating treatments) is insignificant (p≤0.05). Results are presented as mean value±standard deviation (*N*=3)

The initial yeast and mould count in all Kashar cheese samples was 1.95 log CFU/g, which increased by approx. 4 log_10_ during 28 days of storage (Fig. 3). On day 14, the growth of mould and yeast was significantly inhibited (p≤0.05) in the coated cheese samples, particularly those coated with the NaOH-hydrogel. However, by day 28, the coatings did not significantly inhibit mould and yeast growth compared to the control (p≥0.05). While previous studies have demonstrated that chitin-glucan complex inhibits fungal growth (*30*), this effect was not observed when chitin-glucan was applied in hydrogel form to Kashar cheese. This discrepancy could be attributed to the amount of chitin-glucan used, which was optimized for hydrogel formation, rather than its antifungal properties. Higher amounts of chitin-glucan might enhance its antifungal effect, but they were not tested in this study to preserve the hydrogel structure. Chitin-glucan antimicrobial properties are primarily attributed to its ability to interact with microbial cell membranes (*31*). Chitin derivatives, such as chitosan, can disrupt cell wall integrity, inhibit cell membrane functions, and interact with intracellular components like nucleic acids and proteins. These mechanisms hinder microbial growth by increasing cell permeability or by interfering with cellular processes. However, when chitin-glucan is converted into a hydrogel form, the amount and structure of the biopolymer play a crucial role in its effectiveness (*31*). In our study, the lower chitin-glucan amount in the hydrogel may have limited its antifungal effect, as higher amounts are generally more effective in combating microbial growth (*32*).

#### The growth of lactic acid bacteria

**Fig. 4:**
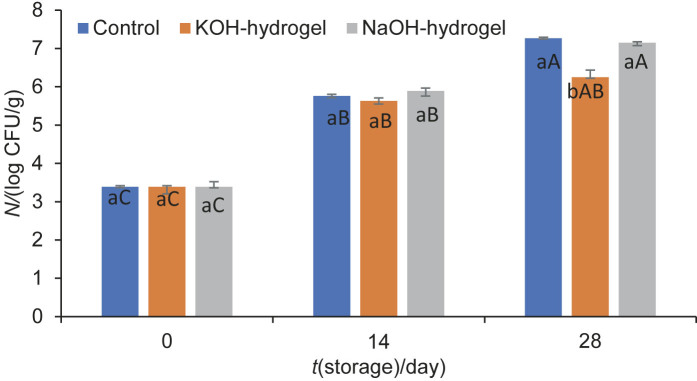
The growth of LAB in Kashar cheese samples coated with chitin-glucan hydrogel prepared in KOH (KOH-hydrogel) and NaOH (NaOH-hydrogel) solvents at 4 °C for 28 days of storage. Control=distilled water. The difference between means denoted by the same uppercase letter (the effect of storage time) and the same lowercase letter (the effect of coating treatments) is insignificant (p≤0.05). Results are presented as mean value±standard deviation (*N*=3)

The number of LAB increased from 3.39 to 6.25 and 7.10 log CFU/g in Kashar cheese samples coated with KOH- and NaOH-hydrogel at the end of the storage, respectively (Fig. 4). Coating with KOH-hydrogel inhibited LAB growth in cheese samples by 1 log, compared to the control and coating with NaOH-hydrogel. The rise in the population of LAB is a crucial factor contributing to increased acidity in the environment, leading to the development of undesirable sour flavour in cheese. The inhibitory effect of the coating with KOH-hydrogel on LAB may have delayed the formation of sour flavour in cheese samples, thereby contributing to an extension in their shelf life.

### Peroxide analysis

**Fig. 5:**
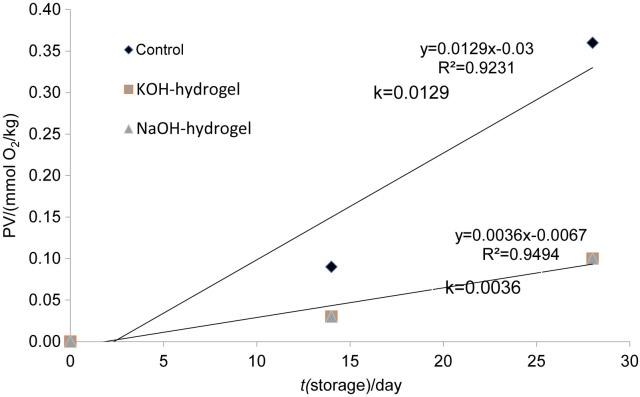
Peroxide value (PV) of Kashar cheese coated with chitin-glucan hydrogel prepared in KOH (KOH-hydrogel) or NaOH (NaOH-hydrogel) solvents during 28 days of storage at 4 °C. The same regression curve was used for both coatings due to statistically similar peroxide formation rates (p≥0.05). Control=samples prepared with distilled water

On the first days of storage, the peroxide value (PV) remained at zero for all cheese samples (Fig. 5). However, on day 14 of storage, both coatings with KOH- and NaOH-hydrogel showed a significant inhibition of peroxide formation in the cheese samples compared to the control group (p≤0.05) (Table 1). No statistically significant difference was found between the effects of coatings with NaOH- and KOH-hydrogel on peroxide values of the cheese samples (p≥0.05).

Peroxide serves as the primary indicator of rancidity resulting from the hydrolysis of fats (*33*). With prolonged storage, the PV typically increases due to enhanced oxidation. Similar studies have shown that the application of hydrophilic coatings can delay fat hydrolysis in foods by slowing down oxygen transfer (*34*-*36*). Consistent with these findings, the hydrogels used in this study may have slowed oxygen transfer and delayed hydrolysis during storage of Kashar cheese.

Chitin-glucan has shown significant antioxidant properties, attributed to its ability to scavenge free radicals and inhibit oxidative processes (*37*). Studies show that β-glucans, which are part of the chitin-glucan complex, can prevent oxidative damage by reducing the effects of reactive oxygen species in biological systems. This antioxidant activity can help protect food products, such as Kashar cheese, from lipid oxidation and rancidity. The hydrophilic nature of chitin-glucan hydrogels likely contributes to delaying oxidative degradation, thereby preserving the quality and extending the shelf life of food products (*31*, *37*).

### Texture analysis

The coating treatment significantly increased the hardness, adhesiveness and gumminess properties and decreased the resilience (p≤0.05) of the cheese samples compared to the control (Table 2). It was observed that the hydrogel coating prepared with NaOH significantly increased the cohesiveness value of the cheese compared to the control, but this value decreased in the hydrogel coating prepared with KOH (p≤0.05). The coated cheese showed a higher hardness value, a decrease in resilience and a difference in adhesive value compared to the control, all of which were associated with higher moisture loss in the coated cheese samples. In similar studies, as in the present study, the relatively higher hardness and stickiness value and the decrease in resilience in coated samples compared to the control were explained by the loss of moisture in the products with hydrogel (*29*, *38*).

**Table 2:** The effect of coatings on the texture properties of fresh Kashar cheese on the last day of storage

Coating	Hardness/MPa	Adhesiveness/(J/m^2^)	Cohesiveness/(J/m^2^)	Resilience/(J/m^3^)	Gumminess/g
Control	(93.50±0.07)^a^	(-42.2±0.5)^a^	(0.6±0.1)^b^	(0.33±0.07)^c^	(58.91±0.07)^a^
KOH-hydrogel	(151.25±0.05)^b^	(-40.8±0.3)^ab^	(0.59±0.09)^a^	(0.25±0.08)^b^	(88.48±0.09)^b^
NaOH-hydrogel	(126.50±0.05)^c^	(-40.1±0.3)^b^	(0.7±0.1)^c^	(0.21±0.04)^a^	(85.39±0.09)^c^

Control=samples prepared with distilled water, KOH- and NaOH-hydrogel=chitin-glucan hydrogel prepared in KOH and NaOH solvents, respectively. The difference between mean values indicated with the same letter in the same column is insignificant (p≥0.05). Results are presented as mean values±standard deviation (*N*=3)

### Colour

L*, a* and b* values of Kashar cheese samples coated with the hydrogels obtained using chitin-glucan nanofibre are shown in Table 3. The L* value indicates the colour transition from light to opaque, a* value from green to red and b* value from blue to yellow. The highest L* value was 87.4±0.9 in the control samples and the lowest value was 72.01±0.09 in cheese samples coated with hydrogel prepared with KOH during storage. The application of coating significantly decreased the L* value of the cheese samples (p≤0.05). During colour measurement, the L* value approaches 0 for dark and matte colours and 100 for white and bright colours. In this study, since the natural colour of chitin-glucan, used as a polymer in the coating, is close to black, it plays an important role in making the colour appear slightly darker and more matte.

**Table 3:** Effect of different coatings on L*, a* and b* values ​​of Kashar cheese during storage at 4 °C

Colour parameter	*t*(storage)/day	Coating		
		Control	KOH-hydrogel	NaOH-hydrogel
L *	0	(87.4±0.9)^ABb^	(78.4±0.4)^Aab^	(79.2±0.2)^Aa^
	14	(86.2±0.8)^Bb^	(76.0±0.5)^Aa^	(77.0±0.3)^Aa^
	28	(83.7±0.3)^Aab^	(72.01±0.09)^Ab^	(73.0±0.3)^Ba^
a *	0	(2.6±0.2)^Cc^	(3.1±0.2)^Ba^	(3.5±0.3)^Cb^
	14	(1.07±0.03)^Ab^	(1.91±0.02)^Aa^	(2.7±0.3)^Aa^
	28	(0.4±0.2)^Bc^	(1.74±0.06)^Bb^	(1.34±0.03)^Ba^
b *	0	(17.0±1.0)^Aa^	(16.9±1.0)^Aa^	(16.0±0.9)^Aa^
	14	(15.6±0.8)^Bb^	(16.3±0.1)^Aa^	(15.27±0.07)^Ab^
	28	(16.1±0.3)^ABa^	(16.1±0.1)^Aa^	(15.4(0.2)^Ab^

Control=samples prepared with distilled water, KOH- and NaOH-hydrogel=chitin-glucan hydrogel prepared in KOH and NaOH solvents, respectively. The difference between mean values denoted by the same uppercase letter in the same column (the effect of storage time) and the same lowercase letter in the same row (the effect of coating treatments) is insignificant (p≤0.05). Results are presented as mean value±standard deviation (*N*=3)

The coating significantly increased a* value (p≤0.05); the highest value was measured in the samples coated with NaOH-hydrogel (Table 3). The lowest value during storage was measured in the control (0.4±0.2).

The coating did not cause a significant change in b* value (p≥0.05). Although there was a decreasing trend in the b* value of the coated samples during storage, the difference between them was insignificant (p≥0.05). In previous studies on Kashar cheese, it was found that different coating types or packaging materials did not have a significant effect on the b* value during storage (*39*, *40*).

### Shelf-life of the Kashar cheese

In this study, zero-order kinetics was selected for shelf-life determination at various temperatures, as it provided the highest R^2^ value compared to other models (*21*). A single regression curve (R^2^) was applied to both coating treatments, as the peroxide formation rates did not differ significantly between them (p≥0.05).

For Kashar cheese, shelf life was further evaluated using the rate constant of peroxide formation, which results from oxidation processes in the cheese. In these calculations, the initial peroxide value was assumed to be 0 mmol O_2_ per kg fat, and deterioration was defined as the formation of 2 mmol O_2_ per kg fat. Based on these assumptions, the shelf life was estimated to be 155 days for uncoated cheese and 555 days for cheese coated with hydrogels.

This approach corresponds to standard methods in food science, where kinetic models are used to predict microbial and chemical changes over time. The use of zero-order kinetics in this study indicates that the rate of deterioration remained constant over the observed period, fitting the linear relationship between the measured parameters and time. The use of peroxide values as an indicator of oxidative stability is widely recognized, particularly for fatty foods like cheese, where lipid oxidation plays a critical role in shelf life. The significant extension of shelf life with hydrogel coating highlights the potential of innovative packaging solutions to improve food preservation and reduce waste.

## CONCLUSIONS

In this study, chitin-glucan extracted from biological waste was successfully converted into a hydrogel and applied as a coating material for Kashar cheese. The hydrogel coatings significantly extended the shelf life of the cheese and maintained its quality during storage. Shelf-life estimation using zero-order kinetics showed that the uncoated cheese had a shelf life of 155 days, while cheese coated with hydrogel obtained using chitin-glucan dissolved either in KOH or in NaOH (denoted as KOH- and NaOH-hydrogel) showed a remarkable extension to 555 days. This extension highlights the effectiveness of the hydrogel coating in reducing oxidation, as indicated by peroxide value. The coatings with KOH- or NaOH-hydrogel effectively reduced oxygen permeability and delayed fat hydrolysis, contributing to better preservation of cheese quality. Among the tested hydrogels, the KOH-hydrogel showed superior inhibitory effects on LAB, which are responsible for souring of cheese. This suggests that the choice of solvent plays a crucial role in enhancing the antimicrobial properties of the hydrogel matrix. Although chitin-glucan is known for its antifungal properties, this effect was not prominent in its hydrogel form, potentially due to the low amount of chitin-glucan or structural changes during hydrogel synthesis. Additionally, hydrogel-coated samples experienced greater water loss than uncoated samples, leading to increased hardness, adhesiveness and gumminess, as well as reduced resilience. This study highlights the potential of chitin-glucan hydrogels as sustainable and functional coatings for extending the shelf life of high-moisture food products. These findings establish a foundation for advancements in biodegradable food packaging systems, addressing global challenges in reducing food waste and improving product quality through innovative materials. Given its biodegradability and low cost, chitin-glucan hydrogel presents a sustainable option for extending the shelf life of high-moisture foods. Future work should optimize formulations and assess their performance under industrial-scale processing.

## Appendix

**Table S1:** The content of biological waste in the medium composition

Fruit and vegetable waste	Zucchini, carrots, cabbage (raw and pickled), tomatoes (raw and canned), peppers (capia, Charleston), eggplant, onions, potatoes, cauliflower, parsley, lettuce, dill, arugula, mint, leek, rosemary, celery, garlic, tangerine, orange, lemon, watermelon, banana, pineapple, sea buckthorn, apple, quince, persimmon, pear, grapefruit, kiwi
Protein waste	Mushrooms, eggs (raw and boiled), cheese (white cheese, cheddar cheese), red meat, beans with meat, chickpeas with meat
Polysaccharide waste	Bread (wholegrain, rye, white, whole wheat), cake, pancakes, bagel, pastry (with dill, cheddar, tomato), raisin bun, croissant (chocolate and plain), sugar bun, mooncake

**Table S2:** The chemical composition of biological waste in the medium

Component	Protein	Polysaccharide	Fruit and vegetable
	*w*/%		
Total dry matter	(37.93±0.02)^b^	(76.4±0.5)^a^	(13.05±0.04)^c^
Protein	(19.0±0.3)^a^	(13.0±0.5)^b^	(1.7±0.1)^c^
Fat	(14.65±0.07)^a^	(9.2±0.6)^b^	(1.35±0.06)^c^
Ash	(0.66±0.01)^a^	(0.64±0.04)^a^	(0.31±0.06)^b^
Polysaccharide	(2.54±0.02)^b^	(3.48±0.03)^a^	(2.11±0.03)^c^
Total sugar	(4.92±0.01)^c^	(8.6±0.02)^a^	(6.57±0.01)^b^
